# Supplementary material for: Marine-Derived Bioactive Peptides Self-Assembled Multifunctional Materials: Antioxidant and Wound Healing
Source: Antioxidants (Basel). 2023 May 31;12(6):1190. doi: 10.3390/antiox12061190 (PMC10295045; doi:10.3390/antiox12061190)
Supplement: Supplementary file 1 [file antioxidants-12-01190-s001.zip › antioxidants-2416921-supplementary.pdf]

Marine-Derived Bioactive Peptides Self-Assembled Multifunctional Materials: Antioxidant and Wound Healing

Dingyi Yu [1], Shenghao Cui [1], Liqi Chen [1], Shuang Zheng [1], Di Zhao [1], Xinyu Yin [1], Faming Yang [1,*] and Jingdi Chen [1,2,*]

1 Marine College, Shandong University, Weihai 264209, China; 202000810156@mail.sdu.edu.cn (D.Y.); 202000810226@mail.sdu.edu.cn (S.C.); 201900810011@mail.sdu.edu.cn (L.C.); 202100810142@mail.sdu.edu.cn (S.Z.); 201900810068@mail.sdu.edu.cn (D.Z.); 202100810117@mail.sdu.edu.cn (X.Y.)
2 Shandong Laboratory of Advanced Materials and Green Manufacturing, Yantai 265599, China
* Correspondence: yangfm0123@163.com (F.Y.); jdchen@sdu.edu.cn (J.C.); Tel.: +86-6315688303 (J.C.)

Supplementary materials

1. *Physicochemical analysis of CAPs*

To detect the surface hydrophobicity of CAPs prepared in different pH environments and concentrations, the ANS (1-anilino-8-naphtalene-sulfonate; RHAWN, #R003056-25G) fluorescent probe method was used. An 8 mM ANS solution was pre-pared in pH 7.0 PBS and stored away from light. Serial dilutions of each sample were prepared using deionized water in the range of 5–80 mg. To each well of the 200 μL sample, 4 μL of 8 mM ANS was added, and the mixture was incubated in the dark at 25°C for 45 min. The resulting samples were analyzed in a black 96-well plate, and the fluorescence intensity was measured using a well plate reader (ENSPIRE, Thermo Fisher, USA) with excitation at 365 nm and emission at 484 nm. The sample solution without ANS and the pH 7 PBS solution were used as blank. The hydrophobicity (initial slope S0) was determined based on a linear regression analysis (Excel) of fluorescence intensity versus protein concentration.

The samples were analyzed by Ultimate 3000 system (ThermoFisher Scientific, USA) equipped with a reversed-phase ReproSil-Pur C18-AQ resin column (150 μm×15 cm, 1.9 μm, 100 Å, Dr. Maisch GmbH, Germany). Samples (5 μL) were injected into the LC-MS/MS system and eluted with water (A) and acetonitrile (B) at 600 nL/min under the following conditions: 4%–8% B for 2 min, 8%–28% B for 43 min, 28%–40% B for 10 min, 40%–95% B for 1 min and 95% B for 10 min. The CAPs were identified using Q Exactive™ Hybrid Quadrupole-Orbitrap™ Mass Spectrometer (Thermo Fisher Scientific, USA). The mass spectrometry was performed at 300–1800 m/z in positive ionization. The CAPs analysis was performed under the following conditions: Resolution: 17,500, AGCtarget: 1e5, MaximumIT: 60ms, TopN: 20, NCE/steppedNCE: 27.

2. *In vitro healing activity verification system*

2.1 Antioxidant activity of CAPs

The antioxidant activity of CAPs was estimated using Ferric Reducing Ability of Plasma (FRAP), 1,1-Diphenyl-2-picrylhydrazyl (DPPH), and 2,2′-azinobis (3-ethylben-zothiazoline 6-sulfonate) (ABTS) assays.

The FRAP method was performed according to the protocol of Total Antioxidant Capacity Assay Kit with FRAP (Beyotime Institute of Biotechnology, China). The FRAP reagent was freshly prepared before use. 5 μL of diluted samples and 180 μL of FRAP reagents were added to a 96-well plate. After incubation 37°C for 3-5 min, the absorbance at 593 nm was measured using a microplate reader (VersaMax, Molecular Devices, USA).

The 1,1-Diphenyl-2-picrylhydrazyl (DPPH) radical scavenging ability of CAPs radicals were assayed referring to previous studies. Mixtures of 2 mL of DPPH solution (0.1 mM in 95% methanol) and 2 mL of samples with various concentrations were prepared, and the ascorbic acid as the positive control. After incubation at room temperature without exposure to light for 30 min. Using a microplate reader (Versa-Max, Molecular Devices, USA), an absorbance of 517 nm was measured.

The ABTS free radical scavenging assay was based on a classical method as previously reported. The ABTS free radical cation solution was prepared by mixing ABTS solution (0.7 mM) with 2.45 mM potassium persulfate at room temperature overnight for 16 h. Next, the ABTS free radical cation solution was diluted with PBS to the absorbance of 0.70 ± 0.05 at 734 nm. Afterward, 5.0 μL aliquot of samples was added to 200 μL of diluted ABTS+ solution, which were incubated at room temperature for 10 minutes before they were collected. An absorbance measurement at 734 nm was performed using a microplate reader (VersaMax, Molecular Devices, USA).

2.2 Cell proliferation and cytotoxicity assay

The cell proliferation and cytotoxicity were measured with human keratinocytes (HaCaTs were purchased from Suzhou Beina Chuanglian Biotechnology Co., Ltd. (Suzhou, China)). At 37°C, DMEM/F12 medium containing 10% fetal bovine serum (FBS) and 5% CO_2 was used as a culture medium for the HaCaTs. The cell proliferation ability was tested using a Cell Counting Kit-8 (CCK-8) assay. Briefly, HaCaTs (2×10^5 cells/well) were added in 96-well plates for 4 h to form a monolayer. Then, HaCaTs were treated with positive drug (Human FGF-basic, Pepro Teck Co., Ltd., USA) and 10 μL of CAPs with various concentrations (400, 200, 100, 50, 25, 12.5, 6.25 μg/mL), respectively. After culturing for 24 h, 100 μL of CCK8 diluted with DMEM/F12 medium was added to each well and incubated for 1 h. Lastly, an absorbance measure was taken at 450 nm using a microplate reader (VersaMax, Molecular Devices, USA).

The cytotoxicity assay was carried out using an AO/EB Staining Kit (Sangon Biotech, Shanghai). A 72-well plate was seeded with HaCaTs and incubated for 24 h. Two washes with PBS were then performed. To stain the cells, 5 μL of AO Staining Solution and 5 μL of EB Staining Solution were added to the cell suspension and incubated for 5 min in the dark at room temperature. The mixture was washed with PBS. The staining cells were observed using a florescent microscope (Axio Observer, ZEISS, Germany).

3. *Molecular docking*

The protein-ligand binding capacity of CAPs was predicted using AutoDock-Tools-1.5.6. The crystal structure of the EGFR PDB accession code: 3POZ at 1.50 Å resolution was obtained from the RCSB Protein Data Bank and defined as the receptor. The structure of selected CAPs was constructed using ChemDraw software (CambridgeSoft Co., Ltd，USA). Next, the water molecules and organic ligands were removed, and hydrogens were added to the model. Grid boxes were set to enclose the entire proteins with the following coordinates: x = 126, y = 126, z = 126. And the docking procedure was performed using the following parameters: Number of

GA Runs: 10, Maximum Number of evals: long, Number of Active Torsion in Ligand: 15. The docking results were analyzed using PyMoL package.

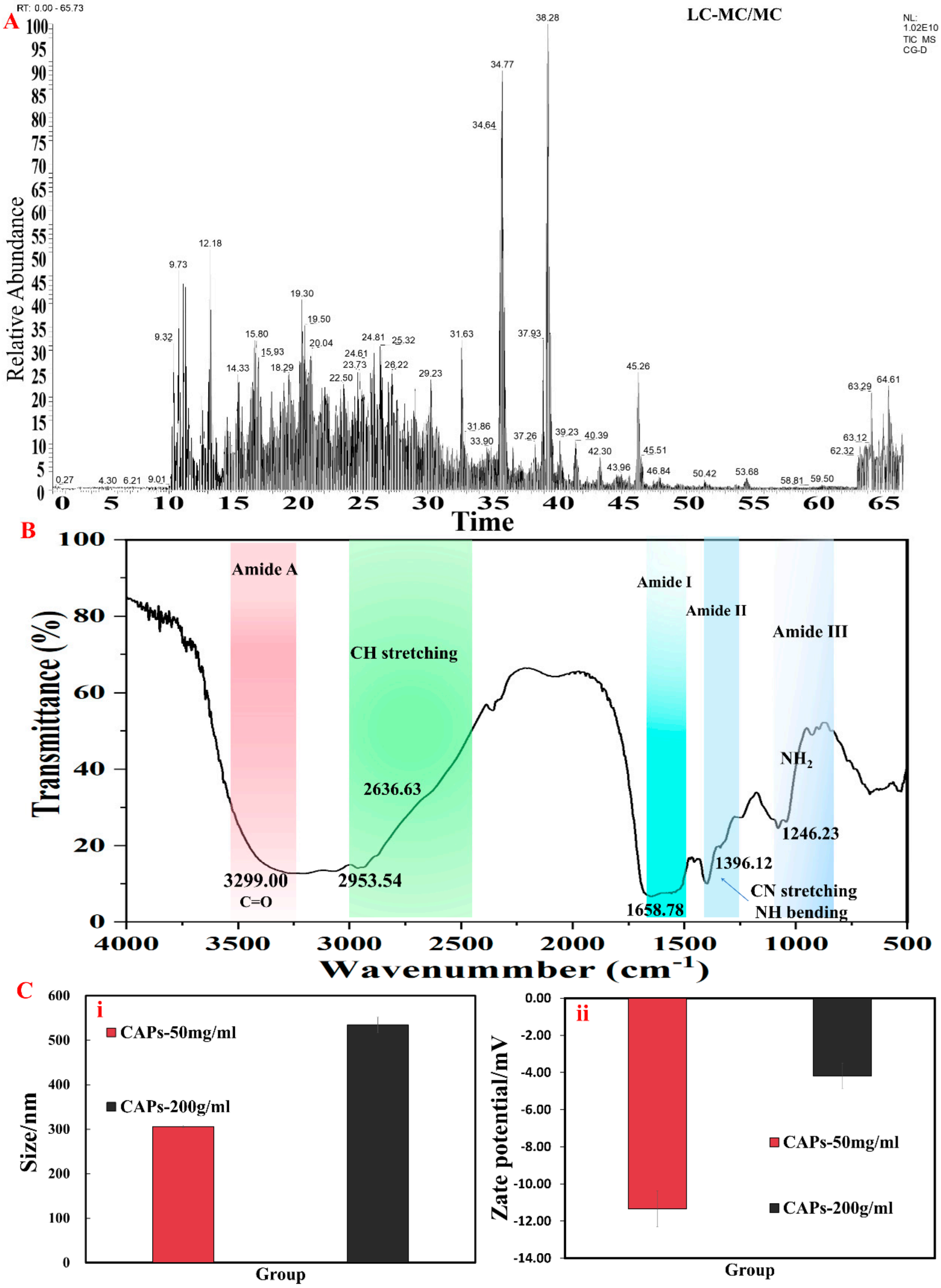

Figure S1. Physicochemical analysis of CAPs. (**A**) Total ion chromatogram of CAPs. (**B**) FTIR spectra of CAPs. (**C**) Effects of concentration on the averaged particle size (i) and zeta potential (ii) of CAPs.

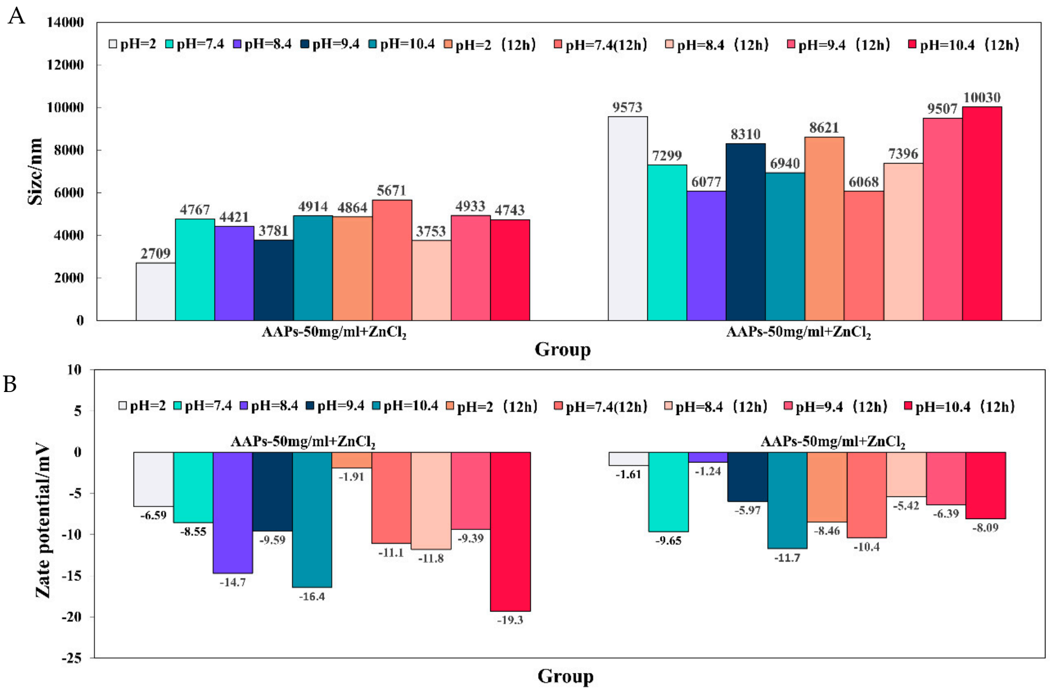

Figure S2. Effect of Zn^{2+} addition on averaged particle size. (**A**) and zeta potential (**B**) of CAPs (0 h and 12 h) with different pH values.

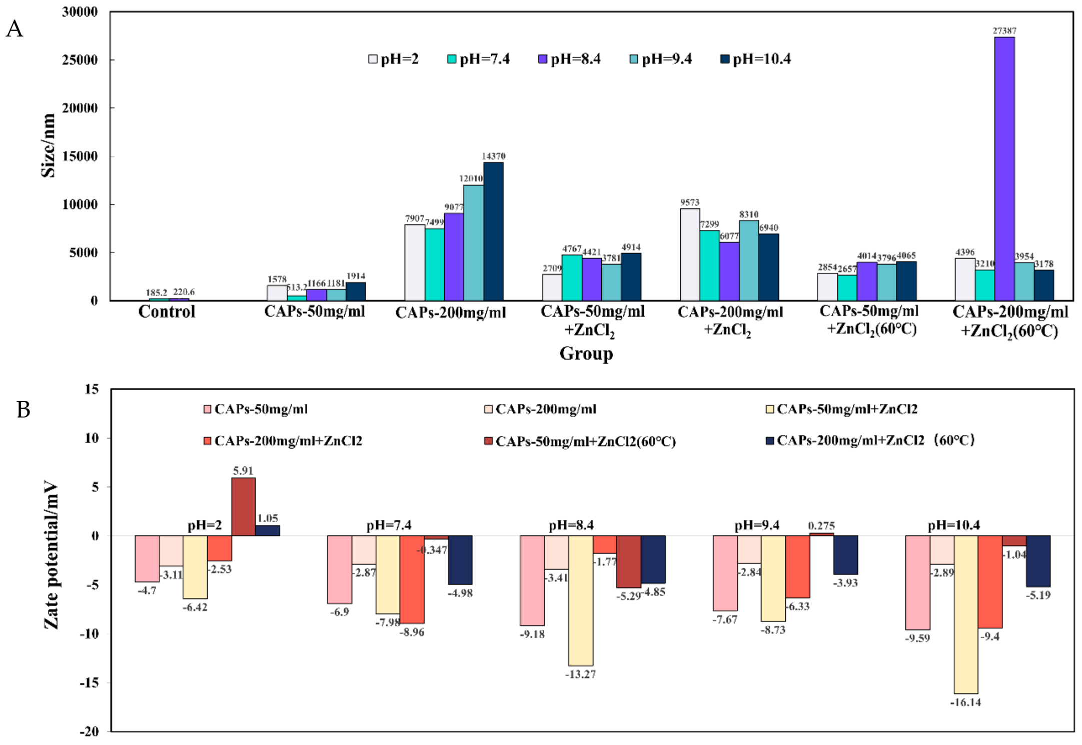

Figure S3. Effect of temperature on the particle size/potential of CAPs. (**A**) Effect of Zn^{2+} addition on averaged particle size of CAPs with different pH values at room temperature and 60°C. (**B**) Effect of Zn^{2+} addition on zeta potential of CAPs with different pH values at room temperature and 60 °C.

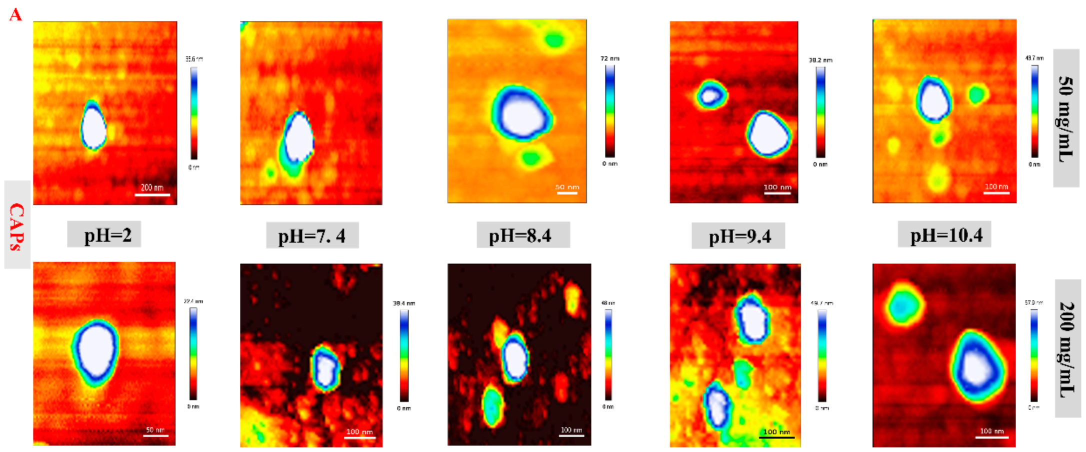

Figure S4. AFM images of various concentrations of CAPs at different pH conditions.

Table S1. The secondary structure content calculated from CD spectras of various concentrations of CAPs at different pH conditions.

System	α-Helix (%)	β-Sheet (%)	β-Turn (%)	Random coil (%)
CAPs-50mg/mL-pH 2.0	0.0	76.9	0.0	23.1
CAPs-50mg/mL-pH 7.4	0.0	77.2	0.0	22.8
CAPs-50mg/mL-pH 8.4	0.0	76.6	0.0	23.4
CAPs-50mg/mL-pH 9.4	0.0	77.7	0.0	22.3
CAPs-50mg/mL-pH 10.4	0.0	76.7	0.0	23.3
CAPs-200mg/mL-pH 2.0	0.0	76.8	0.0	23.2
CAPs-200mg/mL-pH 7.4	0.0	76.5	0.0	23.5
CAPs-200mg/mL-pH 8.4	0.0	77.4	0.0	22.6
CAPs-200mg/mL-pH 9.4	0.0	76.6	0.0	23.4
CAPs-200mg/mL-pH 10.4	0.0	65.5	0.0	34.5
